# Rapid selection of environmentally friendly layered alkaline-earth metal phosphates as solid lubricants using crystallographic data

**DOI:** 10.1038/s41598-018-34478-5

**Published:** 2018-11-01

**Authors:** Xiaosheng Zhang, Wenxing Niu, Yingjing Dai, Hong Xu, Jinxiang Dong

**Affiliations:** Research Institute of Special Chemicals, College of chemistry and chemical engineering, Taiyuan University of Technology, Taiyuan, 030024 Shanxi P.R. China

## Abstract

Lubricating technologies are essential for saving energy and identifying effective, environmentally friendly layered materials that meet the demands of solid lubricants is an important direction. Herein, we probed the relationships between load-carrying capacity and crystal structure. The results showed that increasing the sliding resistance of interlayers using corrugated layers increased the load-carrying capacity of layered solid lubricants. This finding expands on the traditional lubrication mechanism of layered materials. Following the rules, rapid selection of layered potassium magnesium and calcium phosphates (K-LMP and K-LCP) as effective solid lubricants was achieved using crystallographic data and strict filtering criteria. In order to prepare materials suitable for lubrication, the synthesis of K-LMP and K-LCP was optimised. These materials could be utilised in the food, textile or marine machinery industries in the future. The developed method could successfully guide the synthesis of application-oriented materials.

## Introduction

With the rapid development of human civilisation, energy consumption is constantly increasing. To meet this growing need, major strategies include increasing the effective use of fossil fuels, developing new alternative energies, and reducing consumption of conventional energy^1,2^. It has been reported that one third of the world’s energy resource consumption is wasted through friction and wear^3^, and the effective use of lubricants offers a potential route to increasing energy use efficiency.

Solid lubricants are essential for heavy impact/load operations since they provide wear protection and enhance load-carrying performance. Molybdenum disulfide (hereafter referred to as MoS_2_) is the predominant material used as a solid lubricant due to its lamellar crystalline structure with weak van der Waals-type bonds between planes. This substance can prevent metal on metal contact under high-load conditions^4–6^. However, molybdenum is a scarce resource that represents a source of heavy metal pollution, and the lubricating ability of MoS_2_ deteriorates over time due to oxidation to MoO_3_. Thus, it is important to develop other layered materials as effective and environmentally friendly solid lubricants.

Previous research in this area has mainly focused on magnesium silicate hydroxide, layered double hydroxides, layered disodium silicates, layered zirconium phosphates, and some others^7–15^. Among these, magnesium silicate hydroxide and layered double hydroxides possess good friction-reduction and anti-wear properties, but fail to improve the load-carrying capacity of lubrication oil and grease. Layered disodium silicates and layered zirconium phosphates exhibit good anti-wear, friction-reduction and load-carrying performance. The tribological properties of these layered materials have been evaluated under different test conditions, but the relationships between crystal structures and tribological properties remain poorly understood.

In the present work, the tribological properties of the above-mentioned layered materials were investigated under identical test conditions, and the relationships between crystal structures and tribological properties were analysed based on the experimental data. We found that layered disodium silicate and layered zirconium phosphate possess better tribological properties than magnesium silicate hydroxide and layered double hydroxides, especially under higher load. The lubricating action is not only due to weak van der Waals-type bonds between planes, but also the corrugated layer, which generates slide resistance and acts as a buffer that facilitates a higher load-carrying capacity.

Guided by these finding, we selected environmentally friendly layered potassium magnesium and calcium phosphates with corrugated layers using the periodic table and the Inorganic Crystal Structure Database (ICSD). Synthesis conditions were established by varying the magnesium/calcium source, the molar ratio of raw materials, and the reaction temperature. A four-ball tester was employed to evaluate the tribological properties of biodegradable coconut oil calcium-based grease using the synthesised layered phosphates as solid lubricants. The results revealed that the layered potassium magnesium and calcium phosphates significantly increase the load-carrying capacity.

## Results

### Probing the relationships between load-carrying capacity and crystal structure

We tested MoS_2_, graphite, magnesium silicate hydroxide (Mg_6_Si_4_O_10_(OH)_8_; hereafter referred to as MgSH), magnesium aluminium layered double hydroxides ([Mg_0.66_Al_0.33_(OH)_2_](Cl)_0.33_∙nH_2_O; hereafter referred to as MgAl-LDH), layered zirconium phosphate (α-Zr(HPO_4_)_2_∙H_2_O; hereafter referred to as α-ZrP) and layered disodium silicate (β-Na_2_Si_2_O_5_; hereafter referred to as β-LDS) as inorganic layered solid lubricants. We then evaluate their friction and wear performance as additives in PAO8 lithium-based grease (hereafter referred to as lithium-based grease) under identical test conditions using a four-ball tester, which is commonly employed in friction tests. To investigate the lubricating properties of the six layered materials as lubricant additives, they were dispersed in pure lithium grease as described in the Methods section. The maximum non-seizure load (*P*_B_ value) of the additives were determined to represent the load-carrying performance. The anti-wear (WSD value, the wear scar diameter) and friction-reducing (COF value, the coefficient of friction) properties were also studied under different applied loads and temperatures.

**Figure 1:**
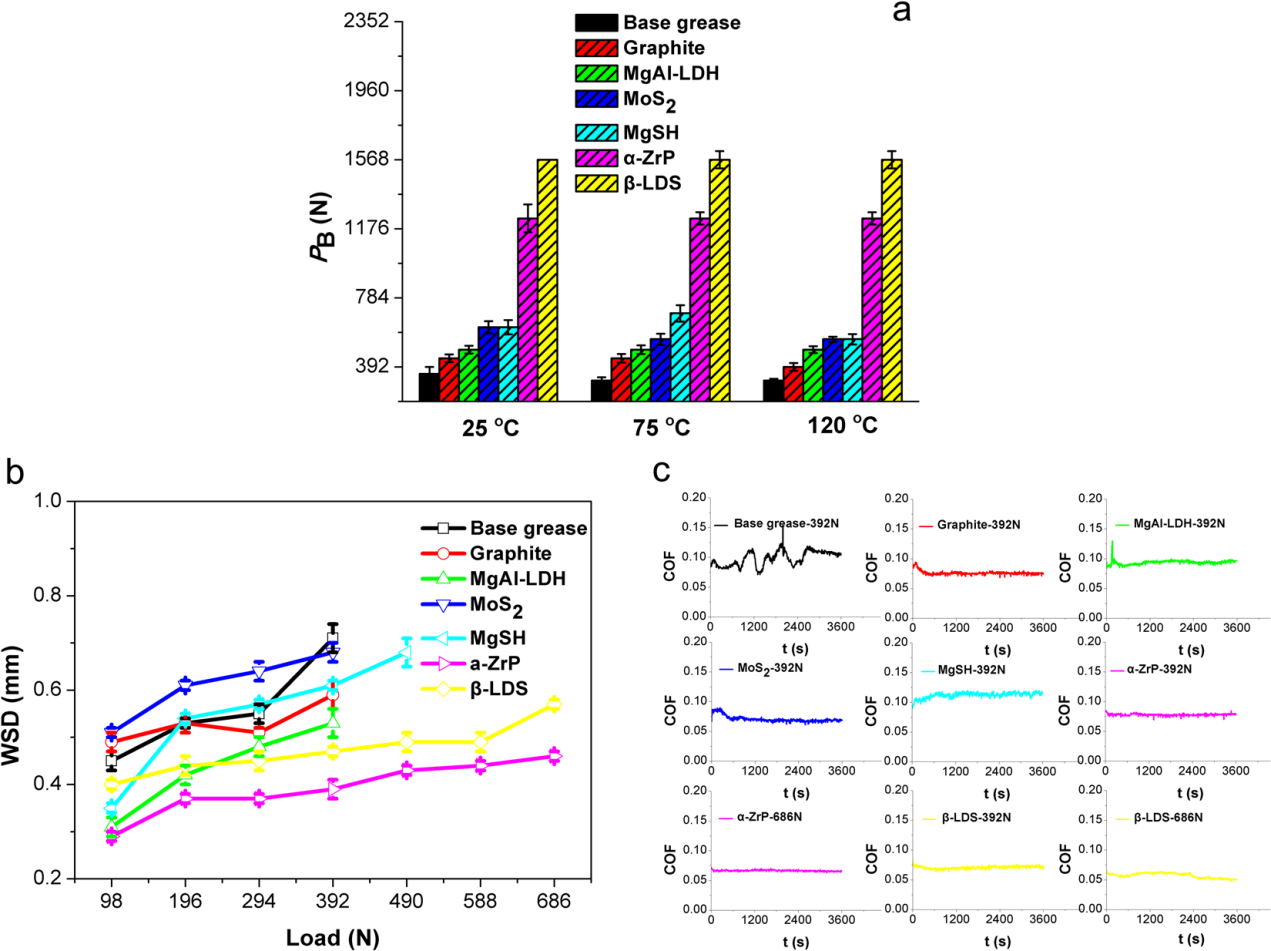
Friction and wear performance of various typical layered lubricant materials as additives in PAO8 lithium-based grease. (**a**) *P*_B_ values at different temperatures (25, 75 and 120 °C). (**b**) WSD values under different loads (from 98 to 686 N). (**c**) Dynamic friction curves under standard load (392 N) and maximum applied loads of β-LDS and α-ZrP (686 N). Test conditions: four-ball test, *P*_B_: 1770 rpm, 10 s; WSD/COF: 1200 rpm, 3600 s, 75 °C.

*P*_B_ values as a function of temperature are displayed in Fig. 1a. All additives proved capable of improving the *P*_B_ value of pure lithium grease, and 5.0 wt.% β-LDS and 5.0 wt.% α-ZrP performed better than the other four additives at increasing the load-carrying capacity of base grease at different temperatures (25, 75 and 120 °C). Since the additive amounts rang from 1.0 wt.%, 3.0 wt.% and 7.0 wt.% at different temperatures (25, 75 and 120 °C), the *P*_B_ values tended to be similar to the amount at 5.0 wt.% (Supplementary Figure S1). WSD values as a function of load at 75 °C are plotted in Fig. 1b. The highest load with no seizure was recorded with β-LDS and α-ZrP at 686 N, and the second highest was with MgSH (490 N), whereas only 392 N was recorded for MoS_2_, graphite, MgAl-LDH and pure lithium grease. Under the highest applied load, WSD values for β-LDS and α-ZrP were 0.57 and 0.46 mm, respectively, lower than other materials under their maximum applied load. This trend held true on temperatures between 25 °C and 120 °C (Supplementary Figure S2). The corresponding worn surfaces of β-LDS and α-ZrP, assessed using a 3D optical profiler, were narrower and shallower than those of the other additives under their maximum applied load (Supplementary Figure S4). These results showed that β-LDS and α-ZrP have better anti-wear properties under a higher applied load. From the dynamic friction curves in Fig. 1c, the friction coefficient of β-LDS and α-ZrP were 0.057 and 0.067, respectively, and remained steady throughout the maximum applied load tests (686 N). The friction coefficients for MgSH, MoS_2_, MgAl-LDH and graphite were 0.112, 0.071, 0.094 and 0.076, respectively, under the applied load (392 N). Consistent with the dynamic friction results at 25 °C and 120 °C (Supplementary Figure S3), β-LDS and α-ZrP displayed better friction reduction performance than the other additives at higher load. Indeed, β-LDS and α-ZrP not only showed excellent load-carrying capacity, but also improved the anti-wear and friction-reducing properties of lithium grease.

**Figure 2:**
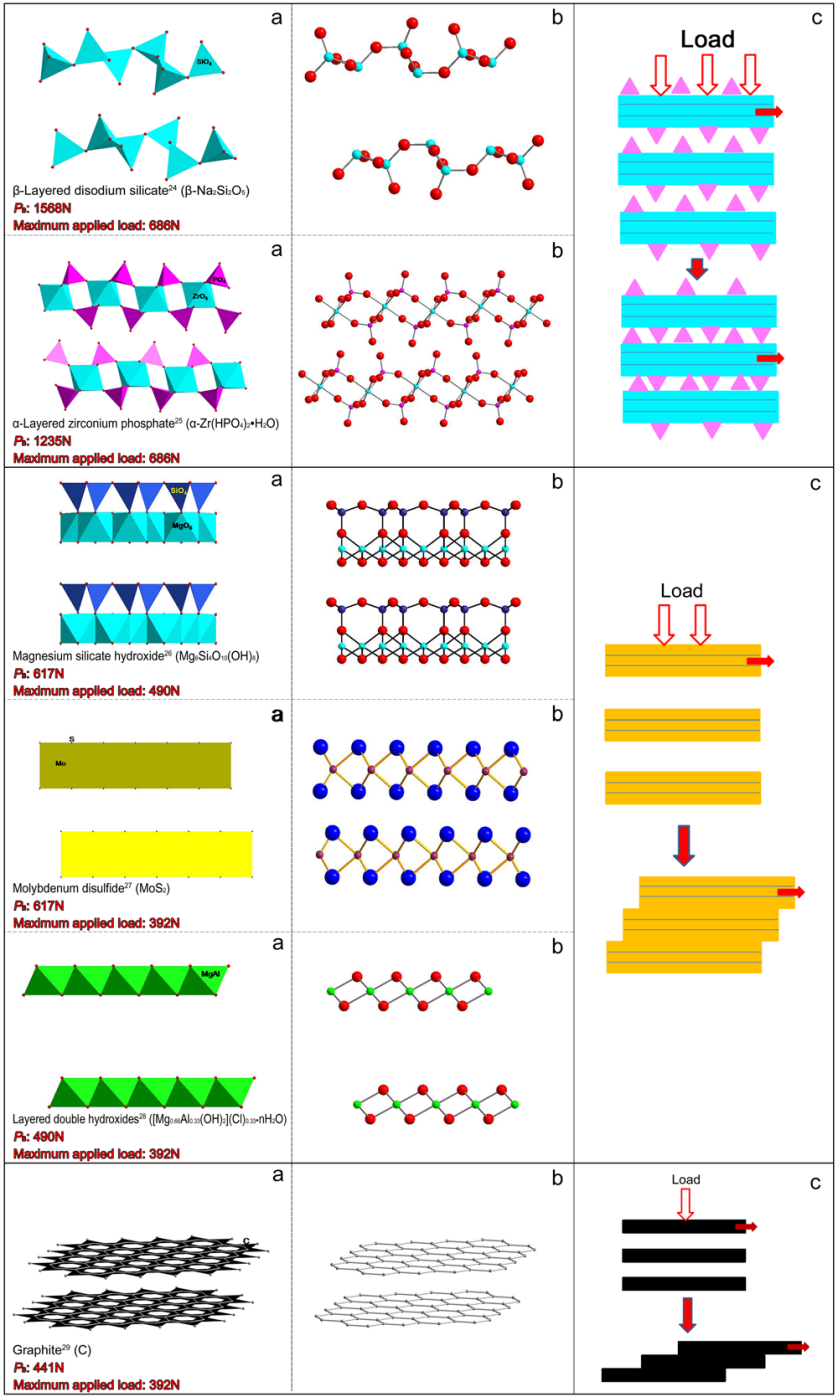
Summary of crystal structure data and load-carrying mechanisms for some typical layered materials. (**a**) Polyhedral conformation^24–29^. (**b**) Ball and stick model. (**c**) Load-carrying mechanism. Test conditions: *P*_B_: 25 °C, 1770 rpm, 10 s; Maximum applied load: 75 °C, 1200 rpm, 3600 s.

In the layered materials, the atoms within a layer are strongly bound to each other, while the layers themselves are relatively far apart and held together via weak van der Waals forces between layers^16^. As a result, the lubricity of the layered materials is due to planes of atoms sliding easily over one another. To better understand why β-LDS and α-ZrP possess good tribological properties, we obtained crystal structure data, *P*_B_ values, and maximum applied loads for the six layered materials (Fig. 2). The crystal structure data revealed that graphite has a monolayer carbon atom layer in which the atoms lie in almost truly planar basal planes. Meanwhile, the atom layers of the other five materials are arranged in multilayers. Atoms in the outer layer board of MoS_2_, MgSH and MgAl-LDH are mostly on the same plane, while the layer boards of β-LDS and α-ZrP are crumpled up. According to the *P*_B_ data under short (10 s) test conditions, the maximum non-seizure loads for β-LDS, α-ZrP, MoS_2_, MgSH, MgAl-LDH and graphite were 1568, 1235, 617, 617, 490 and 441 N, respectively. Meanwhile, under longer (3600 s) friction-wear test conditions, the maximum applied loads for β-LDS, α-ZrP, MoS_2_, MgSH, MgAl-LDH and graphite were 686, 686, 490, 392, 392 and 392 N, respectively. From the above results, layered materials with multilayer atom plates appear to support higher carrying loads than graphite with its monolayer carbon atom layer. β-LDS and α-ZrP have a corrugated layer, and displayed the best load-carrying capacity and had better anti-wear properties, especially under a higher applied load. When subjected to an impact force, the corrugated layer may induce slide resistance and provide a buffer to maintain a higher load-carrying capacity. Therefore, materials with a corrugated layer may perform better at improving the load-carrying capacity. Based on this assumption, an investigation was launched to rapidly select environmentally friendly layered phosphates as solid lubricants using crystallographic data.

### Selection and synthesis of layered magnesium and calcium phosphates

**Figure 3:**
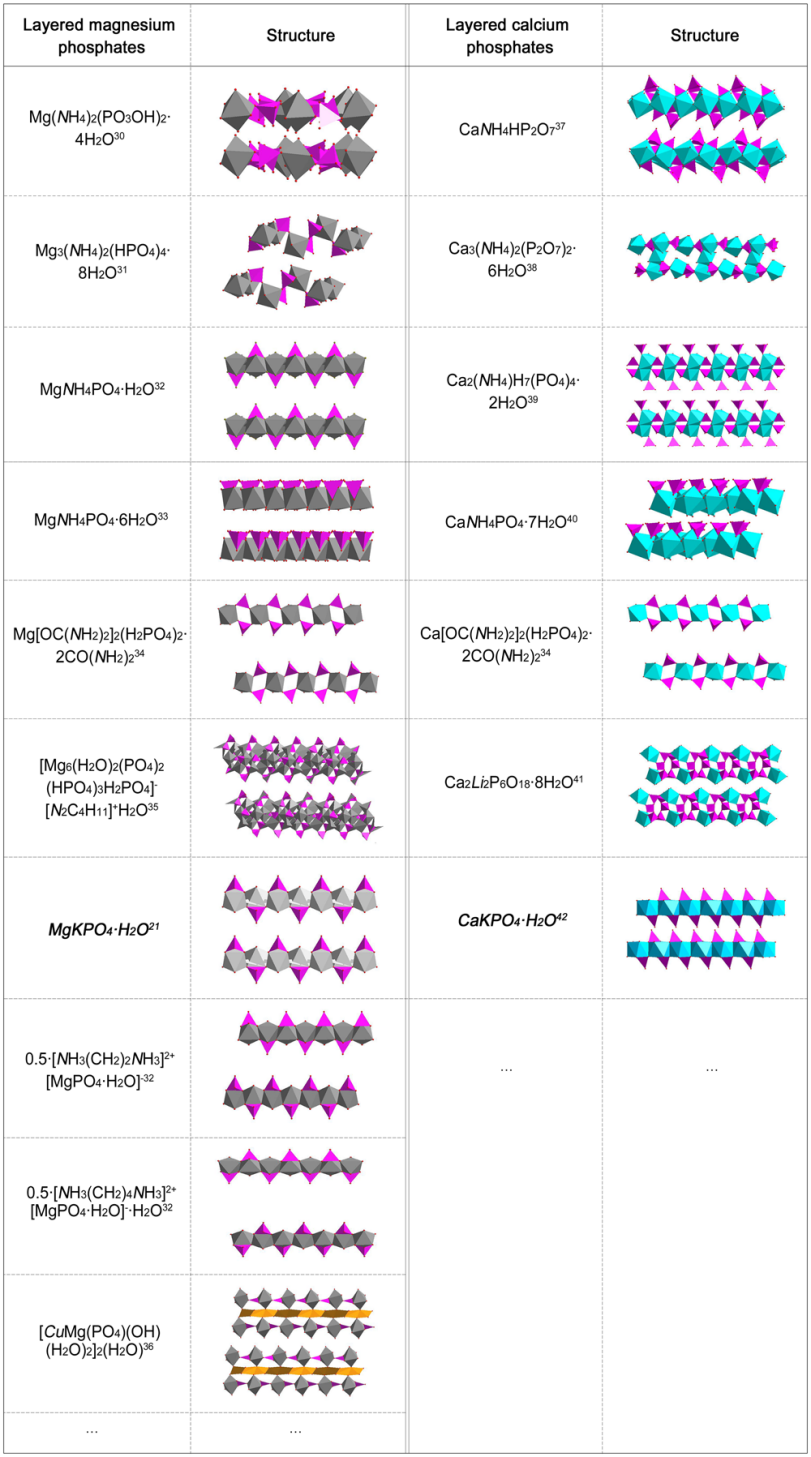
Families of layered magnesium and calcium phosphate compounds. (Interlayer cations are omitted)^30–42^.

**Figure 4:**
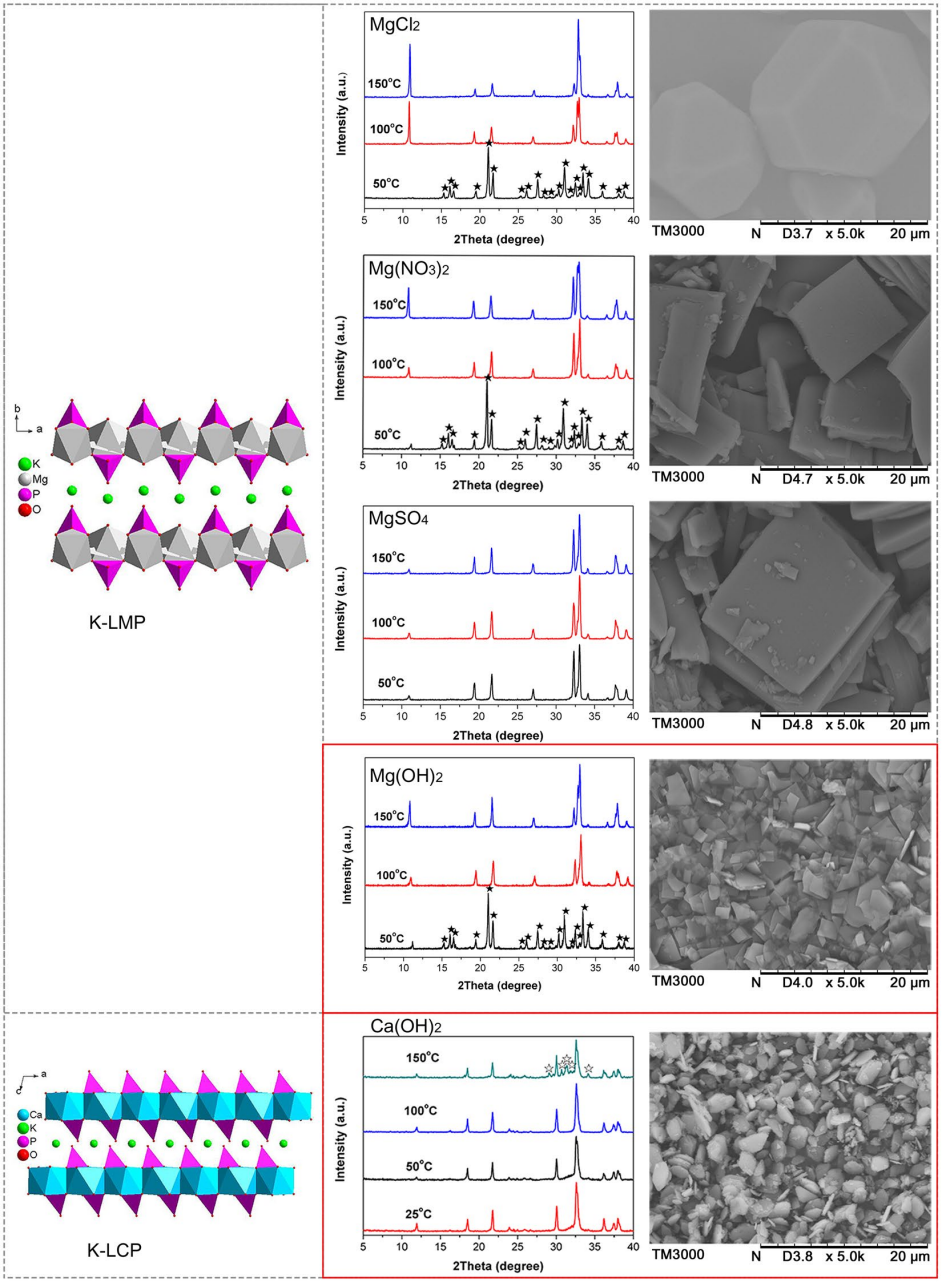
Controlling the particle size of layered magnesium and calcium phosphates. (★ = MgKPO_4_∙6H_2_O; ☆ = impurity phase).

With increasing awareness of the importance of environmental protection and desire to replace unsafe products, the demand for effective and green lubricants is growing^17,18^. Thus environmentally friendly layered phosphates are in demand as solid lubricants. According to the periodic table, alkali metals sodium and potassium, and alkaline earth metals magnesium and calcium are environmentally friendly and abundant resources. Sodium and potassium phosphates are water soluble, so their crystal structures may be unstable in damp environments. However, magnesium and calcium phosphates are not only green, plentiful and water-insoluble; they also perform important physiological roles, and are safe for use in foods, medicines and cosmetics^19,20^. Thus, layered magnesium and calcium phosphates with corrugated layers were tested in the present work. A search of the Inorganic Crystal Structure Database (ICSD) identified 17 layered magnesium and calcium phosphate compounds with corrugated layers (Fig. 3). Among these, materials intercalated with the uneconomic lithium, the heavy metal copper, and environmentally-unfriendly amine compounds (including inorganic ammonium and organic amine compounds) were excluded. Two representative layered potassium magnesium and calcium phosphates (MgKPO_4_∙H_2_O and CaKPO_4_∙H_2_O, hereafter referred to as K-LMP and K-LCP, respectively) with similar crystal structures were eventually selected. These are composed of inorganic phosphate corrugated layers separated by charge-compensating potassium ions (Fig. 4).

Layered potassium magnesium and calcium phosphates were prepared as described below. K-LMP was first synthesised using a MgCl_2_-K_2_HPO_4_-H_2_O system as described previously^21^. The resulting crystals ranged in size from 10−20 μm, and the particle size was too large and therefore unsuitable for lubrication^22,23^. In order to obtain a suitable particle size, various magnesium sources were chosen for K-LMP synthesis (Fig. 4). K-LMP platelets with a particle size of ~2.0 μm were obtained by replacing magnesium chloride with magnesium hydroxide. The low solubility and high alkalinity of magnesium hydroxide may play a key role in controlling particle size. Detailed synthesis conditions are shown in Supplementary Table S1. By changing the synthesis conditions, K-LMP can be prepared using a feed ratio range P/Mg = 2.0–2.2, H_2_O/Mg = 15–30, and a temperature range 100–150 °C in 24 h.

Because an artificial synthesis of K-LCP has not been reported, synthesis conditions were explored by testing different calcium salts as a calcium source using a KOH-Ca salt-K_2_HPO_4_-H_2_O system. Detailed synthesis conditions are shown in Supplementary Table S2. It proved difficult to obtain K-LCP using calcium chloride, calcium nitrate or calcium sulfate as a calcium source. K-LCP platelets of ~2.0 μm in size were successfully synthesised using a KOH-Ca(OH)_2_-K_2_HPO_4_-H_2_O system (Fig. 4). Further analysis with calcium hydroxide as the calcium source resulted in the preparation of K-LCP with a feed ratio range KOH/Ca = 0.5–0.7, P/Ca = 0.75–1.25, and H_2_O/Ca = 20–40 at a temperature range of 25–100 °C in 24 h. The experimental and simulated X-ray diffraction patterns and unit cell parameters of K-LMP and K-LCP are shown in Supplementary Figure S5 and Supplementary Table S3.

### Investigating the tribological properties of layered magnesium and calcium phosphates

**Figure 5:**
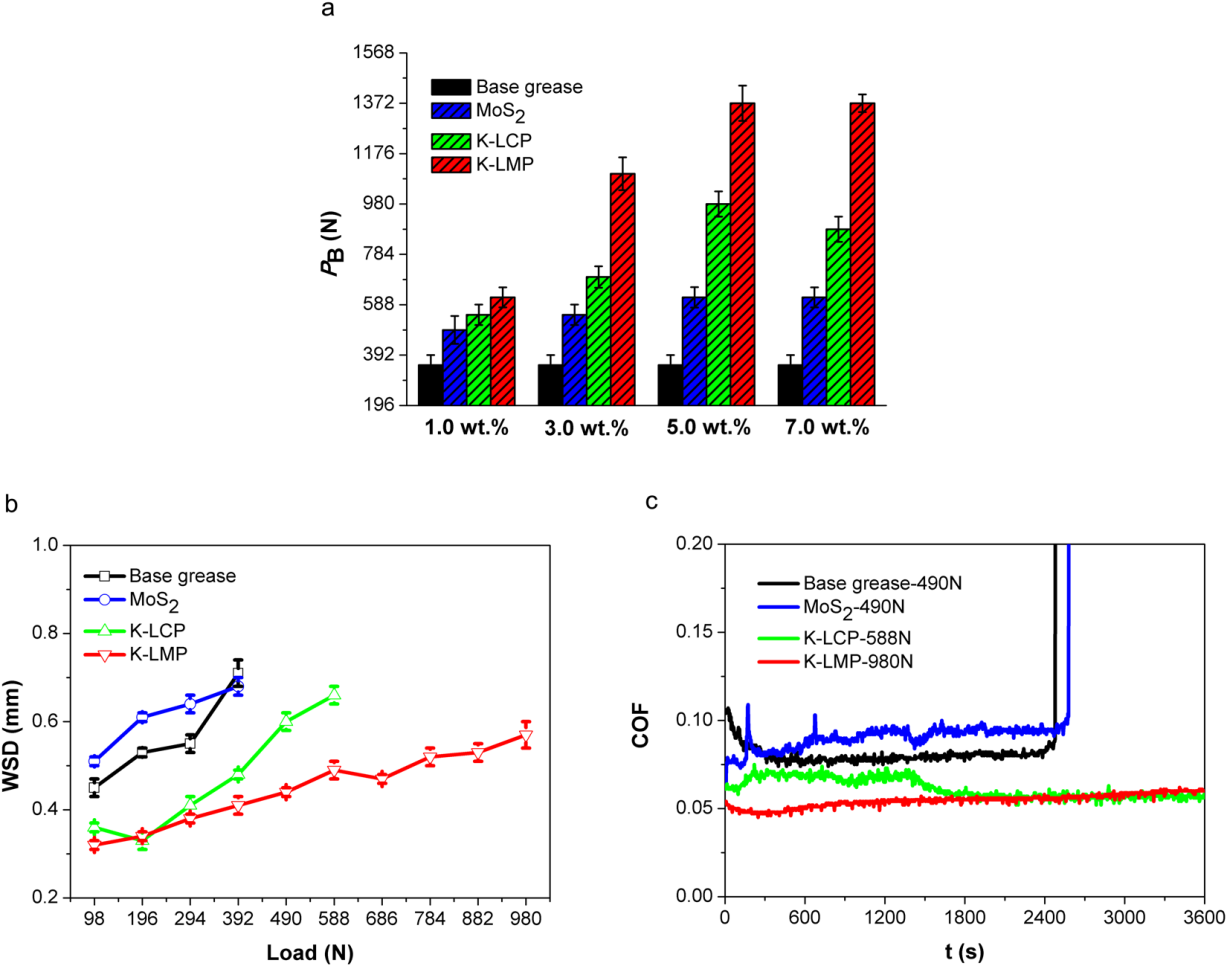
Friction and wear performance of layered potassium magnesium and calcium phosphates as additives in PAO8 lithium-based grease. (**a**) *P*_B_ values with different weight fraction from 1.0 wt.% to 7.0 wt.%. (**b**) WSD values under different loads (from 98 to 980 N). (**c**) Dynamic friction curves under maximum applied loads (K-LMP 980 N, K-LCP 588 N, MoS_2_ 490 N, Base grease 490 N). Test conditions: four-ball test, *P*_B_: 25 °C, 1770 rpm, 10 s; WSD/COF: 75 °C, 1200 rpm, 3600 s.

The tribological properties of the synthesised K-LMP and K-LCP dispersed in lithium grease were evaluated under different concentrations and loads. As shown in Fig. 5a, *P*_B_ values for K-LMP and K-LCP were better than that of MoS_2_ for enhancing the load-carrying capacity of base grease at different additive amounts. Figure 5b shows the variation in WSD values with different applied loads. The highest operating loads for 5.0 wt.% K-LMP grease, 5.0 wt.% K-LCP grease, and 5.0 wt.% MoS_2_ grease were 980, 588 and 392 N, respectively. The WSD values for K-LMP and K-LCP were smaller than that of MoS_2_ under the same load, especially at high loads. Under the highest applied load, the friction curve for K-LMP and K-LCP is stable and smooth, while the curve for base grease and MoS_2_ has numerous fluctuations. These results demonstrate that K-LMP and K-LCP exhibit good performance in terms of anti-wear, friction-reduction and load-carrying capacity.

**Figure 6:**
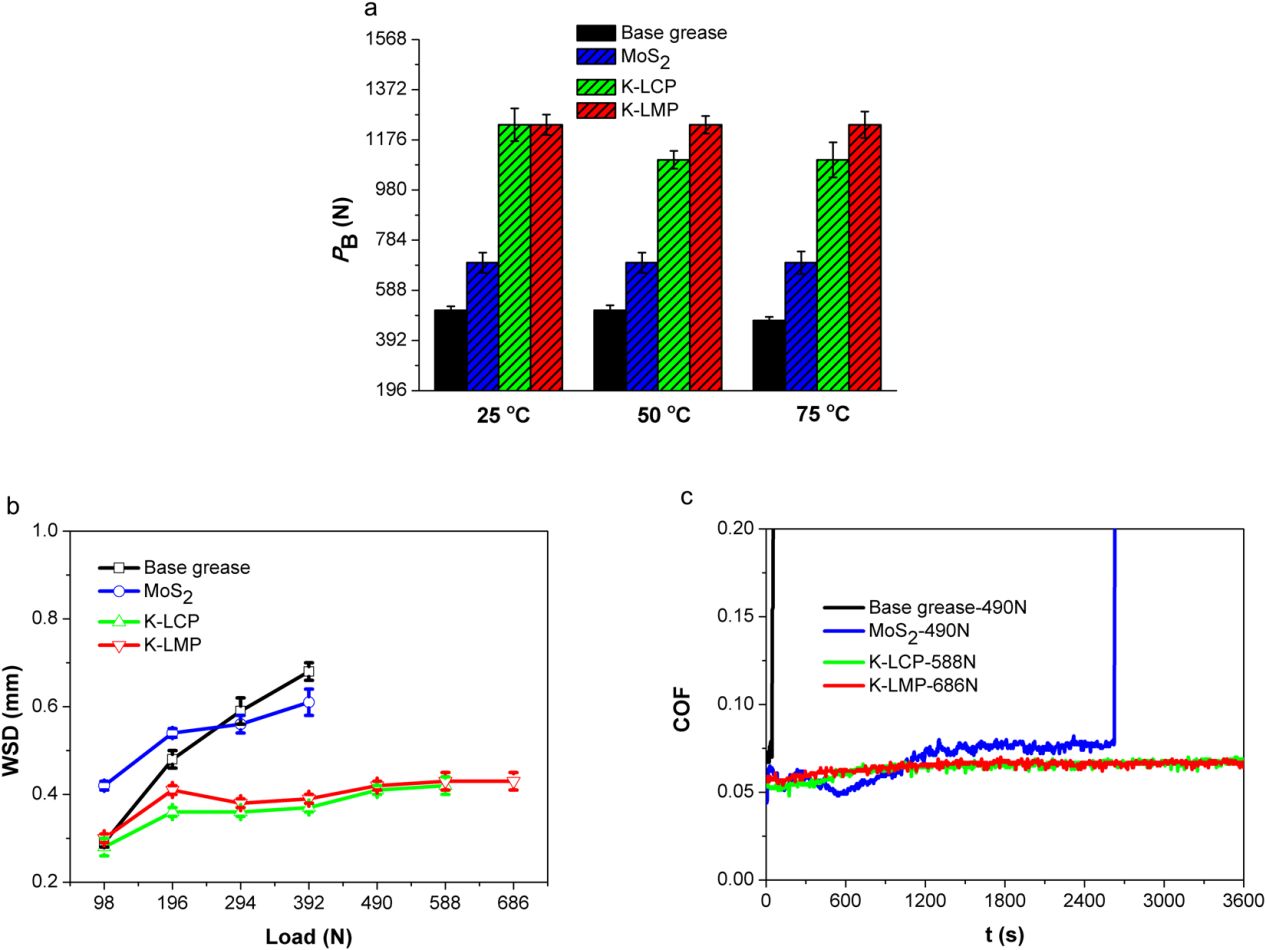
Friction and wear performance of layered potassium magnesium and calcium phosphates as additives in coconut oil calcium-based grease. (**a**) *P*_B_ values at different temperature (25, 75 and 120 °C). (**b**) WSD values under different loads (from 98 to 686 N). (**c**) Dynamic friction curves under maximum applied loads (K-LMP 686 N, K-LCP 588 N, MoS_2_ 490 N, Base grease 490 N). Test conditions: four-ball test, *P*_B_: 1770 rpm, 10 s; WSD/COF: 75 °C, 1200 rpm, 3600 s.

Leakage and other factors result in lubricants polluting the environment both during or after use, and environmentally friendly lubricants are much needed^18^. Lubricants based on environmentally friendly and renewable raw materials and their derivatives are attracting increasing attention for various applications. The general grease composition contains between 65.0 and 95.0 wt.% base oils, 5.0 to 35.0 wt.% thickeners, and 0 to 10.0 wt.% additives. The biodegradability of grease is dependent on the biodegradability of base oils. Herein, coconut oil was chosen as the base oil for investigating the tribological properties of K-LMP and K-LCP as additives in calcium grease. *P*_B_ values at different temperatures (25, 50 and 75 °C) are displayed in Fig. 6a. *P*_B_ values of K-LMP and K-LCP were better than that of MoS_2_ at different temperatures. When adding 5.0 wt.% K-LMP or K-LCP to calcium-based grease, *P*_B_ values increased from 510 to 1235 and 1235 N at 25 °C, from 510 to 1235 and 1098 N at 50 °C, and from 470 to 1235 and 1098 N at 75 °C, respectively. WSD values as a function of load at 75 °C are plotted in Fig. 6b. The highest applied load for K-LMP and K-LCP was 686 N and 588 N, respectively. The corresponding WSD values were 0.43 and 0.42 mm, which are much lower than that of MoS_2_ at different loads, and the dynamic friction coefficients of K-LMP and K-LCP were stable under maximum applied load (Fig. 6b,c). This trend is similar to that of temperature variation at 25 °C and 50 °C (Supplementary Figures S6 and S7). K-LMP and K-LCP as additives in calcium-based grease also displayed good load-carrying, anti-wear and friction-reducing capacities.

**Figure 7:**
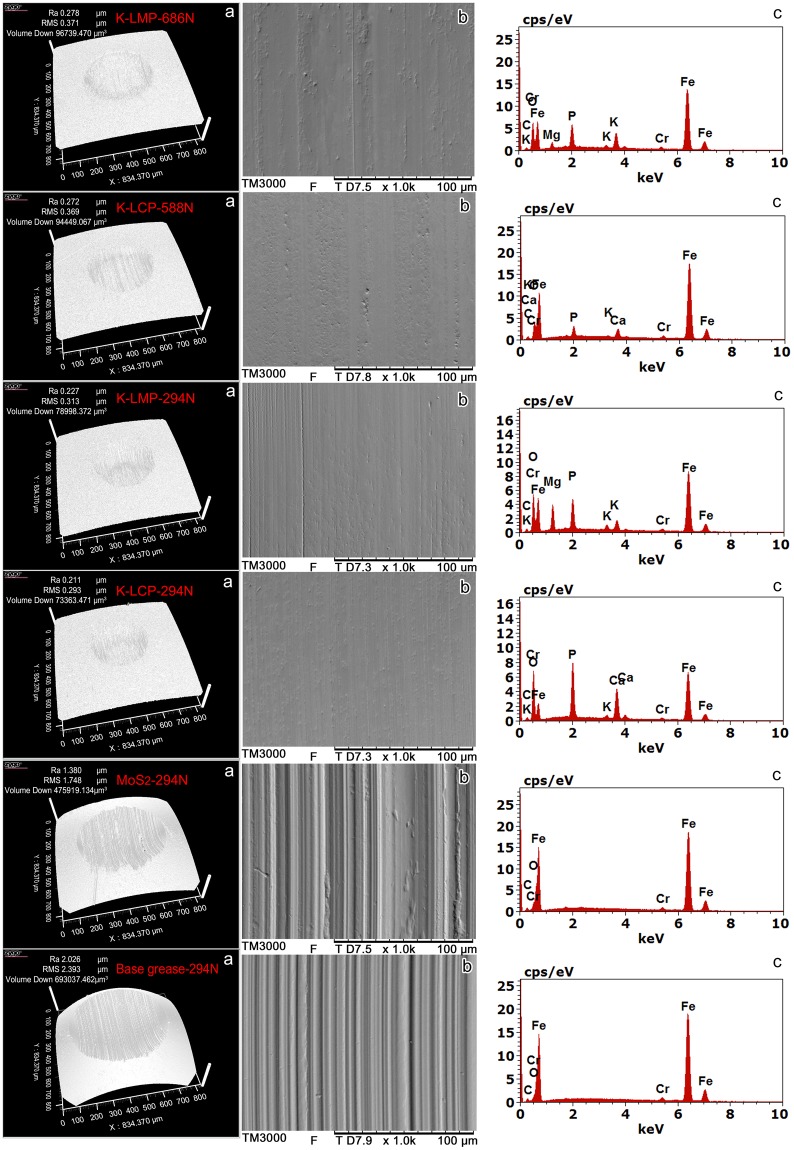
Analysis of the rubbing surface after long run (3600 s) friction-wear testing. (**a**) Non-contact 3D. (**b**) SEM. (**c**) EDS. Details of friction-wear test conditions are as follows: coconut oil calcium-based grease; 75 °C, 1200 rpm, 3600 s. The layered phosphates clearly formed protective films on friction pairs during the friction-wear tests.

Non-contact 3D optical profiling, scanning electron microscopy (SEM) and energy dispersive X-ray spectroscopy (EDS) were employed to characterise worn surfaces of friction pairs **(**Fig. 7). The worn surface of coconut oil calcium-based and grease containing MoS_2_ was severely damaged, with a larger number of deep furrows at an applied load of 294 N. The wear volumes (×10^−4^ mm^3^) of base grease and grease containing 5.0 wt.% MoS_2_, K-LMP and K-LCP were 6.93, 4.76, 0.79 and 0.73, respectively. Moreover, with K-LMP or K-LCP as additive, at the highest applied load the worn surface was smaller with much less furrowing. The wear volumes (×10^−4^ mm^3^) for K-LMP and K-LCP were 0.97 and 0.94, respectively. The EDS results showed that no elements other than those of the friction pair itself (C, O, Fe, Cr) were present on the worn surface of the friction pair lubricated by base grease and grease containing MoS_2_. However, when using grease with K-LMP, Mg, P, and K were present on the worn surface besides Fe, Cr, C, and O, and Ca, P, and K were present when using K-LCP. Supplementary Figure S8 shows the results for the PAO8 lithium-based grease, which are consistent. These results suggest that the layered phosphates with corrugated layers as lubrication additives may form protective films on friction pairs, thereby increasing the lubricating ability, in agreement with the results for β-LDS and α-ZrP in our previous study^8,10^.

To investigate the influence of lubrication on the K-LMP and K-LCP, coconut oil calcium-based grease containing K-LMP or K-LCP after long run (3600 s) friction-wear test at 294 N was adequately diluted with petroleum ether, oleic acid and alcohol to completely remove calcium soap and coconut oil. X-ray diffraction (XRD) and SEM were used to characterise typical solid samples before and after lubrication testing. The powder XRD patterns (Supplementary Figure S9) of K-LMP and K-LCP showed that the diffraction peak positions and diffraction peak intensities are consistent with the original samples. SEM images (Supplementary Figure S10) further confirmed that neither the particle size nor the morphology of K-LMP and K-LCP changed after lubrication testing. The above characterisation results demonstrate that K-LMP and K-LCP as grease additives can remain stable under long-term working conditions.

## Discussion

Effective use of lubricants is an important route for reducing the world’s energy resource consumption. Identifying effective, environmentally friendly layered materials that meet the demands of solid lubricants is therefore of great importance. Herein, we compared the load-carrying capacity of various typical layered lubricant materials (β-LDS, α-ZrP, MgSH, MgAl-LDH, MoS_2_ and graphite) under identical test conditions, and probed the relationships between the load-carrying capacity and crystal structure. We found that layered materials with a corrugated layer performed better in terms of load-carrying capacity. The general view is that the planes of layered materials slide easily over one another, providing resistance to shearing forces and contributing to lubrication. In the present work, we found that increasing the sliding resistance of interlayers using corrugated layers could increase the load-carrying capacity of layered solid lubricants. This finding expands on the traditional lubrication mechanism of layered materials.

In order to identify effective layered solid lubricants, we assessed layered phosphates using crystallographic data, and selected only materials with a corrugated layer that were water-insoluble, environmentally friendly, and available as an abundant resource. Following these rules, two representative layered potassium magnesium and calcium phosphates (K-LMP and K-LCP) were selected from more than 17 layered magnesium and calcium phosphates identified in the Inorganic Crystal Structure Database (ICSD).

In order to prepare materials suitable for lubrication, conditions for synthesising K-LMP and K-LCP were optimised. Regular flake particles with an average particle size of ~2.0 μm were obtained by altering the magnesium/calcium source, the molar ratio of raw materials, and the reaction temperature. The low solubility and high alkalinity of magnesium/calcium hydroxide may play a key role in particle size control.

K-LMP and K-LCP improving the load-carrying capacity, anti-wear and friction-reducing properties of both lithium- and calcium-based grease. Interestingly, a green lubricating system including biodegradable coconut oil, an environmentally friendly calcium thickener, and layered magnesium/calcium phosphates has a better lubricating ability. This suggests layered magnesium and calcium phosphates could be utilised in specific application areas such as the food, textile or marine machinery industries in the future.

In summary, rapid selection of effective layered solid lubricants was achieved using crystallographic data and strict filtering criteria. The established method could successfully guide the synthesis of application-oriented materials in the future.

## Methods

### Materials

The following chemicals were used: amorphous sodium silicate (SiO_2_/Na_2_O mol ratio 1.9–2.3 in the presence of 10–25 wt.% water, Shandong Shengtong Group Share Co,. Ltd.), H_3_PO_4_ (85% in water, aladdin), ZrOCl_2_·8H_2_O (AR, aladdin), NaF (AR, aladdin), MgCl_2_·6H_2_O (AR, aladdin), Mg(NO_3_)_2_·6H_2_O (AR, aladdin), Al(NO_3_)_3_·9H_2_O (AR, aladdin), NaOH (AR, aladdin), KOH (AR, aladdin), Mg(OH)_2_ (AR, aladdin), Ca(OH)_2_ (AR, aladdin), K_2_HPO_4_ (AR, aladdin), MoS_2_ (SCM Industrial Chemical Co. Ltd.), Graphite (Ao Yu Graphite Group), LiOH·H_2_O (AR, aladdin), urea (AR, aladdin), hydrochloric acid (AR, aladdin), anhydrous alcohol (AR, aladdin), stearic acid (AR, aladdin), petroleum ether (Tianjin Fengchuan Chemical Reagent Science and Technology Co. Ltd.), 12-hydroxy stearic acid (Tokyo Chemical Industry Co. Ltd.), polyalphaolefin PAO8 (viscosity of 46.48 mm^2^/s at 40 °C, viscosity index of 146, Chevron Corporation), coconut oil (CP, Aladdin). All chemicals were purchased from commercial sources and used in all syntheses without further purification. Distilled water (H_2_O) was prepared in our laboratory.

### Preparation of β-layered disodium silicate (β-Na_2_Si_2_O_5_, hereafter referred to as β-LDS)

β-LDS was synthesized according to literature^8^, Silicates powder (5 g) was put into a stainless-steel autoclave, and heated at 260 °C for 45 min in a muffle furnace. After cooling the autoclave to room temperature, the solid products were obtained.

### Preparation of α-layered zirconium phosphate (α-Zr(HPO_4_)_2_∙H_2_O, hereafter referred to as α-ZrP)

Based on the typical methods as reported in the literature^10^, α-ZrP was prepared by hydrothermal process in the sealed Teflon-lined stainless steel autoclave (30 mL). Typical reaction conditions are as follows: ZrOCl_2_·8H_2_O (4.5 g, 13.68 mmol), H_3_PO_4_ (3.2 g, 27.69 mmol), NaF (58.8 mg, 1.37 mmol) and H_2_O (5 mL, 277.78 mmol); 180 °C; 36 h.

### Preparation of magnesium silicate hydroxide (Mg_6_Si_4_O_10_(OH)_8_, hereafter referred to as MgSH)

MgSH was prepared by hydrothermal process in the sealed Teflon-lined stainless steel autoclave (30 mL). Typical reaction conditions are as follows: MgCl_2_·6H_2_O (2.6 g, 12.8 mmol), Amorphous sodium silicate (0.67 g, 11.1 mmol), NaOH (2.66 g, 66.6 mmol) and H_2_O (20 mL, 1111.1 mmol); 220 °C; 240 h.

### Preparation of magnesium aluminum layered double hydroxides ([Mg_0.66_Al_0.33_(OH)_2_](Cl)_0.33_∙nH_2_O, hereafter referred to as MgAl-LDH)

MgAl-LDH was prepared by chloride ion ion-exchange on precursor [Mg_0.66_Al_0.33_(OH)_2_](CO_3_)_0.167_∙nH_2_O. A 20 mL mixed saline solution of NaCl (3.0 mol/L) and hydrochloric acid (5.0 mol/L) was added to the precursor under the protection of N_2_ atmosphere, adjusts the pH at 6.0, 100 °C, 4 h. [Mg_0.66_Al_0.33_(OH)_2_](CO_3_)_0.167_∙nH_2_O was prepared through hydrothermal process in the sealed Teflon-lined stainless steel autoclave (30 mL). Typical reaction conditions are as follows: Mg(NO_3_)_2_·6H_2_O (2.56 g, 10.0 mmol), Al(NO_3_)_3_·9H_2_O (1.88 g, 5.0 mmol), urea (3.15 mL, 70.0 mmol) and H_2_O (18.0 mL, 1000.0 mmol); 100 °C; 24 h.

XRD patterns of β-LDS, α-ZrP, MoS_2_, MgSH, MgAl-LDH and Graphite are shown in Supplementary Figure S11.

### Preparation of layered potassium magnesium phosphate (MgKPO_4_·H_2_O, hereafter referred to as K-LMP)

K-LMP was prepared by hydrothermal process in the sealed Teflon-lined stainless steel autoclave (30 mL). Typical reaction conditions are as follows: Mg(OH)_2_ (1 g, 17.24 mmol), K_2_HPO_4_ (6 g, 34.48 mmol) and H_2_O (10 mL, 555.56 mmol); 100 °C; 24 h.

### Preparation of layered potassium calcium phosphate (CaKPO_4_·H_2_O, hereafter referred to as K-LCP)

K-LCP was artificial synthesized for the first time by hydrothermal process in the sealed Teflon-lined stainless steel autoclave (30 mL). Typical reaction conditions are as follows: Ca(OH)_2_ (1 g, 13.51 mmol), K_2_HPO_4_ (5 g, 28.74 mmol), KOH (0.1 g, 1.79 mmol) and H_2_O (10 mL, 555.56 mmol); 50 °C; 24 h.

### Characterization of solid powder samples

The crystallinity and phase-purity of the products was analyzed by powder X-ray diffraction (XRD) on a Rigaku MiniflexII diffractometer with Cu Kα radiation (λ = 1.5418 Å) at 30 kV and 15 mA. The particle size and morphology of materials were investigated by SEM (Hitachi, TM-3000).

### Preparation of lithium-based grease

Lithium-based grease was prepared as follows. First, the predetermined amounts of PAO8, 12-hydroxystearic acid, and stearic acid were added to a grease kettle and the mixture was stirred and heated to 85 °C until it became homogeneous; second, a water slurry of lithium hydroxide initially heated to about 80 °C was slowly added into the above mixture, then heated to 120 °C and kept at this temperature for approximately 3 h until the lithium soap formed. Additional base oil was slowly added into the lithium soap at 90 °C and once again heated to 130 °C until the water completely evaporated. The mixture was kept at 205 °C for 10 min, then cooled to room temperature and ground on a triple-roller mill three times to form the desired grease.

### Preparation of calcium-based grease

Calcium-based grease was prepared as follows. First, the predetermined amounts of coconut oil, 12-hydroxystearic acid and stearic acid were added to a grease kettle and the mixture was stirred and heated to 75 °C until it became homogeneous; second, calcium hydroxide powder was slowly added into the above mixture, then heated to 100–105 °C and kept at this temperature for approximately 2 h until the calcium soap formed. Additional coconut oil was slowly added into the calcium soap at 120 °C for 5–10 min. The mixture was kept at 140 °C for 5–10 min, then transferred to ice water and quenched to room temperature and ground on a triple-roller mill three times to form the desired grease.

To investigate the tribological behavior of β-LDS, α-ZrP, MoS_2_, MgSH, MgAl-LDH, Graphite, K-LMP and K-LCP in the lubricating grease mentioned above, these additives were added at concentrations of 1.0 to 7.0 wt.%. Each variation of lubricating grease was mixed by mechanical stirring and ground three times in a triple-roller mill.

The typical physicochemical properties of these lubricating grease were shown in supplementary Table S4 and S5.

### Tribological Test

The lubricating properties of grease were determined on a four ball tester (Tenkey, MS-10A). The tester is operated with one steel ball, referred to as the “top ball”, under a load rotating against three steel balls held stationary and clamped together for three-point contact. The balls (diameter 12.7 mm, HRc 59–61, Grade 25 EP (Extra Polish)) used in the test were made of GCr15 bearing steel (SAE52100 steel) (composition: 0.95–1.05% C, 0.15–0.35% Si, 0.24–0.40% Mn, 0.027% P, < 0.020% S, 1.30–1.67% Cr, < 0.30% Ni, < 0.025% Cu). Load carrying capacity (*P*_B_ values) were were conducted at a rotating speed of 1770 rpm with 10 s duration at ambient temperature according to the China Petrochemical Standard SH/T 0202-92 (similar to ASTM D2596-97). The four-ball tester was also used to evaluate the anti-wear properties of GCr15 bearing steel self-mated frictional pairs under the lubrication of grease. Lubricating greases were brought to 25, 75 and 120 °C, the rotating speed was 1200 rpm and loads were set to 98, 196, 294, 392, 490, 588, 686, 784 and 882 N for test durations of 3600 s. After each test completion, the WSD values on the stationary balls were measured on an optical microscope with an accuracy of 0.01 mm in the directions parallel and perpendicular to the sliding motion. Three identical tests were performed for an average so as to minimize data scattering. 3D Optical Profiler (ZeGage), SEM (Hitachi, TM-3000) and EDS (Bruker, QUANTAX 70) were employed to analyze the worn surface.

## Electronic supplementary material

Supplementary Information
