# Supplementary material for: Rapid selection of environmentally friendly layered alkaline-earth metal phosphates as solid lubricants using crystallographic data
Source: Sci Rep. 2018 Nov 1;8:16210. doi: 10.1038/s41598-018-34478-5 (PMC6212450; doi:10.1038/s41598-018-34478-5)
Supplement: Supplementary file 1 — Supplementary Information [file 41598_2018_34478_MOESM1_ESM.pdf]

Supplementary Information

Rapid selection of environmentally friendly layered alkaline-earth metal phosphates as solid lubricants using crystallographic data

Xiaosheng Zhang, Wenxing Niu, Yingjing Dai, Hong Xu*, Jinxiang Dong*

Research Institute of Special Chemicals, College of chemistry and chemical engineering, Taiyuan University of Technology, Taiyuan 030024, Shanxi, P.R. China.

Correspondence and requests for materials should be addressed to J.D. (email: dongjinxiangwork@hotmail.com) or to H.X. (email: xuhongwork@126.com).

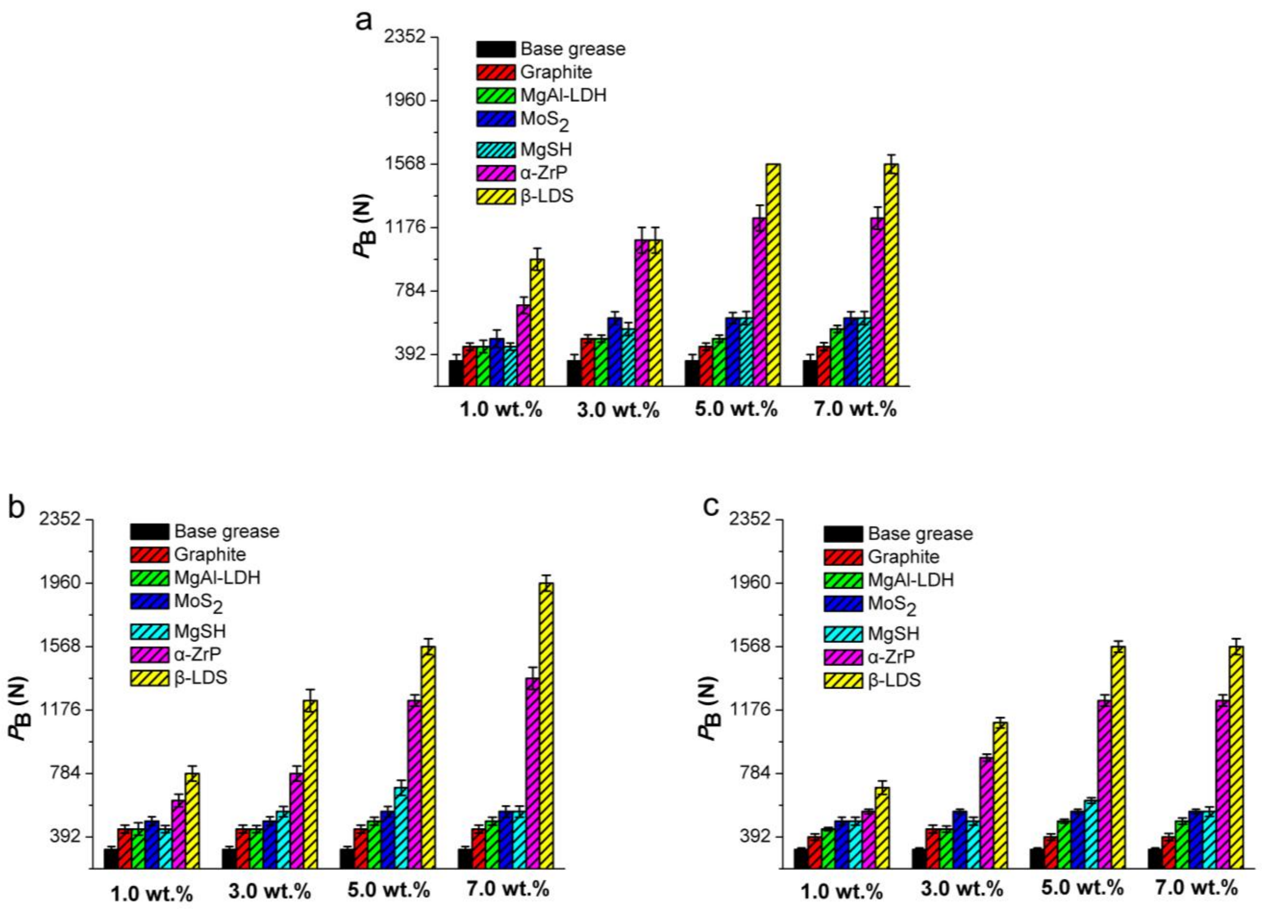

Figure S1. P_B values of lithium-based grease alone and in the presence of additives with different adding amount at different temperatures. (four ball test; 1770 rpm, 10 s; a: 25°C, b: 75°C, c: 120°C)

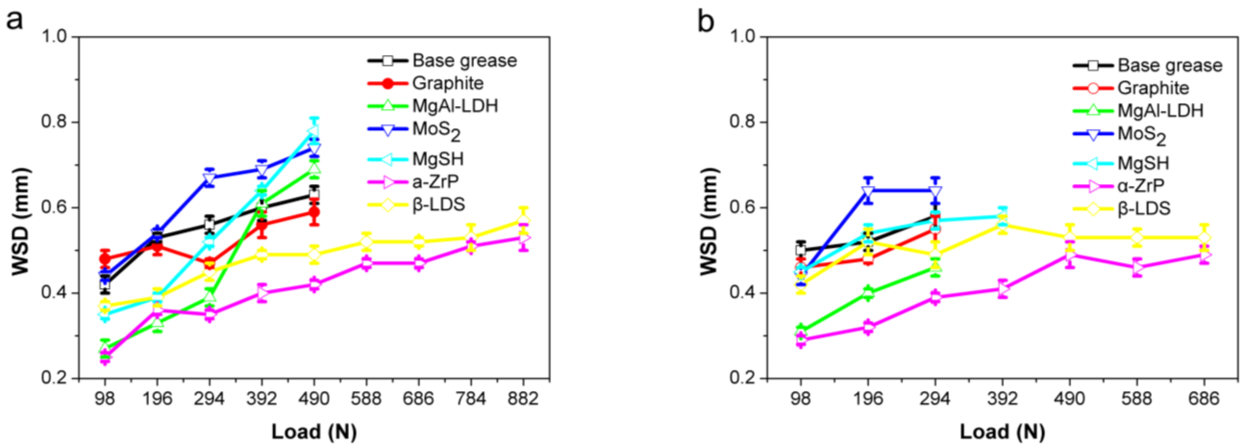

Figure S2. WSD values of lithium-based grease alone and in the presence of 5.0 wt.% additives at different temperatures. (four ball test; 1200 rpm, 3600 s; a: 25°C, b: 120°C)

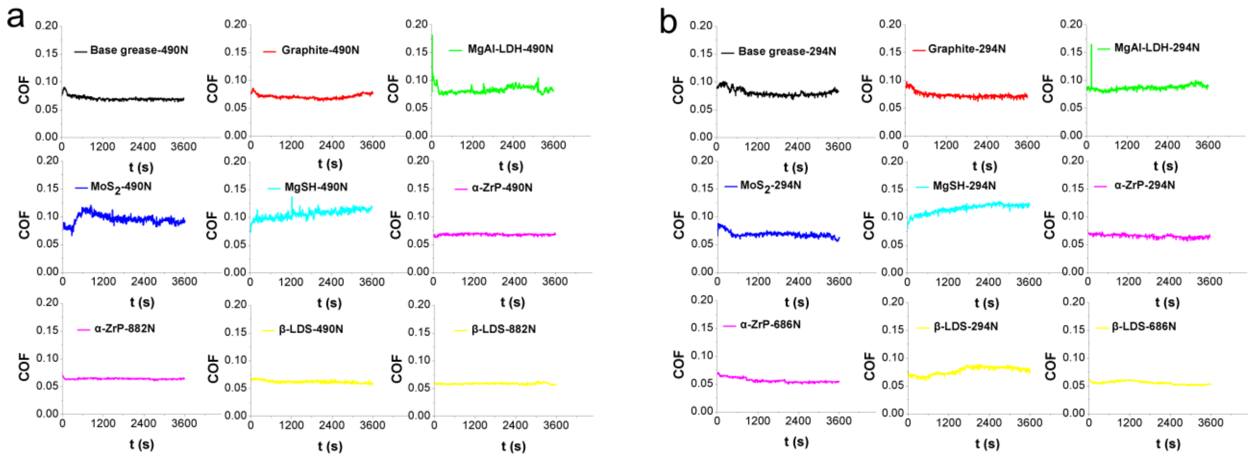

Figure S3. Dynamic friction curves of lithium-base grease alone and in the presence of 5.0 wt.% additives at different temperatures. (four ball test; 1200 rpm, 3600 s; a: 25°C, b: 120°C)

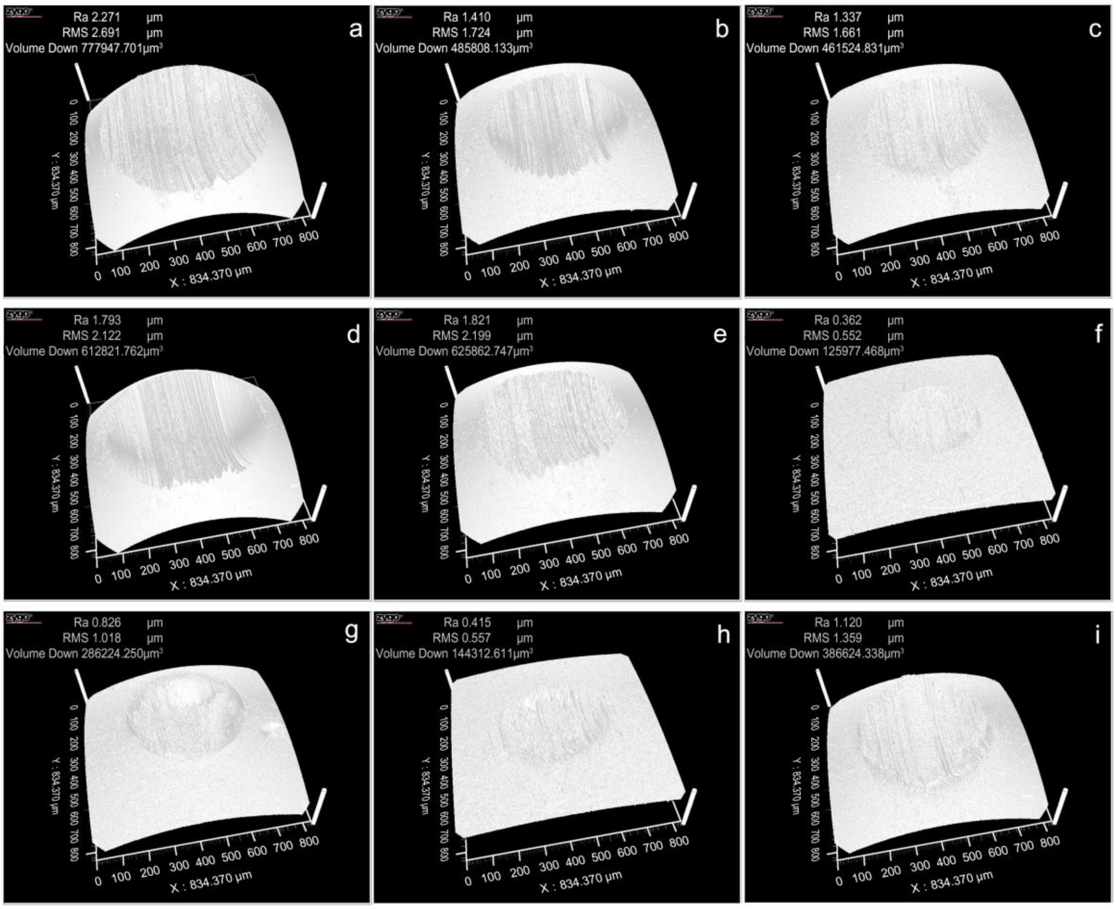

Figure S4. Non-contact 3D optical analysis of the wear scars of lithium-based grease alone and in the presence of 5.0 wt.% additives at 75°C. (a: Base grease-392 N-wear volume 7.78×10^{-4} mm^3, b: Graphite-392 N-wear volume 4.86×10^{-4} mm^3, c: MgAl-LDH-392 N-wear volume 4.62×10^{-4} mm^3, d: MoS_2-392 N-wear volume 6.13×10^{-4} mm^3, e: MgSH-392 N-wear volume 6.26×10^{-4} mm^3, f: α-ZrP-392 N-wear volume 1.26×10^{-4} mm^3, g: β-LDS-392 N-wear volume 2.86×10^{-4} mm^3, h: α-ZrP-686 N-wear volume 1.44×10^{-4} mm^3, i: β-LDS-686 N-wear volume 3.87×10^{-4} mm^3)

Table S1. The synthesis of layered potassium magnesium phosphate using a $Mg(OH)_2$-K_2HPO_4-H_2O system.

magnesium source	molar ratio of raw materials ($P : Mg : H_2O$)	t (h)	T (^oC)	products
$Mg(OH)_2$	1.8:1:30	24	100	K-LMP+$Mg(OH)_2$
	2.0:1:30	24	100	K-LMP
	2.2:1:30	24	100	K-LMP
	2.0:1:15	24	100	K-LMP
	2.0:1:45	24	100	K-LMP+$MgKPO_4 \cdot 6H_2O$
	2.0:1:30	24	50	K-LMP+$MgKPO_4 \cdot 6H_2O$
	2.0:1:30	24	150	K-LMP

Table S2. The synthesis of layered potassium calcium phosphate with different calcium salts as a calcium source using a KOH-Ca salt-K_2HPO_4-H_2O system.

calcium source	molar ratio of raw materials (KOH : P : Ca : H_2O)	T (h)	T (^oC)	products
$Ca(OH)_2$	0.5:0.25:1:20	24	25	$Ca_5(PO_4)_3(OH)$
	0.5:0.75:1:20	24	25	K-LCP
	0.5:1.25:1:20	24	25	K-LCP
	0.5:1.75:1:20	24	25	K-LCP + impurity phase
	0.3:1.25:1:20	24	25	K-LCP + impurity phase
	0.7:1.25:1:20	24	25	K-LCP
	0.9:1.25:1:20	24	25	K-LCP + impurity phase
	0.5:1.25:1:10	24	25	$Ca(OH)_2$+ unknown phase
	0.5:1.25:1:40	24	25	K-LCP
	0.5:1.25:1:80	24	25	unknown phase
	0.5:1.25:1:20	24	50	K-LCP
	0.5:1.25:1:20	24	100	K-LCP
	0.5:1.25:1:20	24	150	K-LCP+ impurity phase
$CaCl_2$	0:1.0:1:60	24	25	$CaPO_3OH{\cdot}2H_2O$
	0:3.0:1:60	24	25	$CaPO_3OH{\cdot}2H_2O$
	0:5.0:1:60	24	25	armorphous phase
	0.5:3.0:1:60	24	25	$CaPO_3OH{\cdot}2H_2O$+$CaPO_3OH$
	1.0:3.0:1:60	24	25	armorphous phase
	0:3.0:1:30	24	25	$CaPO_3OH$
	0:3.0:1:90	24	25	$CaPO_3OH{\cdot}2H_2O$
	0:3.0:1:60	24	50	$CaPO_3OH{\cdot}2H_2O$
	0:3.0:1:60	24	100	$CaPO_3OH$
	0:3.0:1:60	24	150	$CaPO_3OH$
$Ca(NO_3)_2$	0:1.0:1:80	24	25	$CaPO_3OH{\cdot}2H_2O$
	0:3.0:1:80	24	25	$Ca_5(PO_4)_3OH$
	0:5.0:1:80	24	25	$Ca_5(PO_4)_3OH$
	0.3:3.0:1:80	24	25	$Ca_5(PO_4)_3OH$
	0.9:3.0:1:80	24	25	$Ca_5(PO_4)_3OH$
	1.5:3.0:1:80	24	25	$Ca_5(PO_4)_3OH$
	0:3.0:1:40	24	25	$Ca_5(PO_4)_3OH$
	0:3.0:1:120	24	25	$Ca_5(PO_4)_3OH$
	0:3.0:1:80	24	50	$Ca_5(PO_4)_3OH$
	0:3.0:1:80	24	100	$Ca_5(PO_4)_3OH$

	0:3.0:1:80	24	150	$Ca_5(PO_4)_3OH$
$CaSO_4$	0:1.0:1:90	24	25	$CaPO_3OH{\cdot}2H_2O$
	0:3.0:1:90	24	25	$Ca_5(PO_4)_3OH$
	0:5.0:1:90	24	25	$Ca_5(PO_4)_3OH$
	0.3:3.0:1:90	24	25	$Ca_5(PO_4)_3OH$
	0.9:3.0:1:90	24	25	$Ca_5(PO_4)_3OH$
	1.5:3.0:1:90	24	25	$Ca_5(PO_4)_3OH$
	0:3.0:1:30	24	25	$Ca_5(PO_4)_3OH$
	0:3.0:1:60	24	25	$Ca_5(PO_4)_3OH$
	0:3.0:1:120	24	25	$Ca_5(PO_4)_3OH$
	0:3.0:1:90	24	50	$Ca_5(PO_4)_3OH$
	0:3.0:1:90	24	100	$Ca_5(PO_4)_3OH$
	0:3.0:1:90	24	150	$Ca_5(PO_4)_3OH$

Table S3. Simulated and experimental unit cell parameters of the typical layered potassium magnesium and calcium phosphates refined with PCW.

Sample	a	b	c
K-LMP (Experimental)	5.6016 Å	8.2819 Å	4.8199 Å
K-LMP (Simulated)	5.573 (2) Å	8.231 (2) Å,	4.790 (2) Å
K-LCP (Experimental)	7.5922 Å	8.1763 Å	7.6634 Å
K-LCP (Simulated)	7.5834 (9) Å	8.1568 (1) Å	7.6541 (8) Å

a: PCW is a GUI (Graphical User Interface) - based program that is used to represent the unit cells and calculated powder pattern.

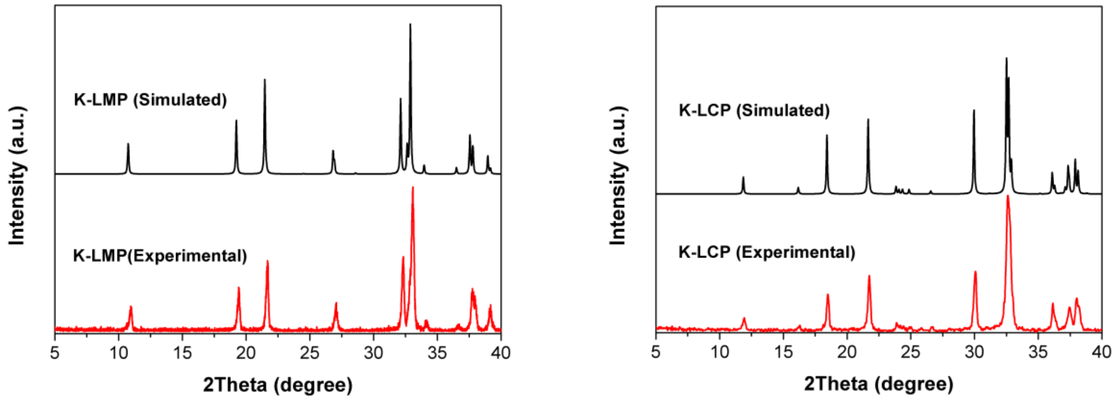

Figure S5. Simulated and experimental X-ray diffraction patterns of the typical layered potassium magnesium and calcium phosphates.

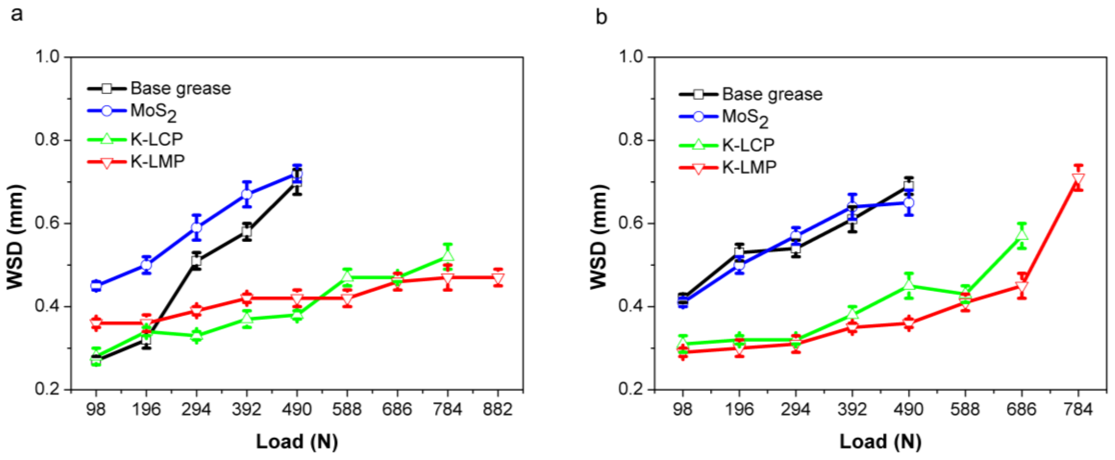

Figure S6. WSD values of coconut oil calcium-based grease alone and in the presence of 5.0 wt.% layered potassium magnesium and calcium phosphates at different temperatures. (a) 25°C. **(**b) 50°C. Test conditions: 1200 rpm, 3600 s.

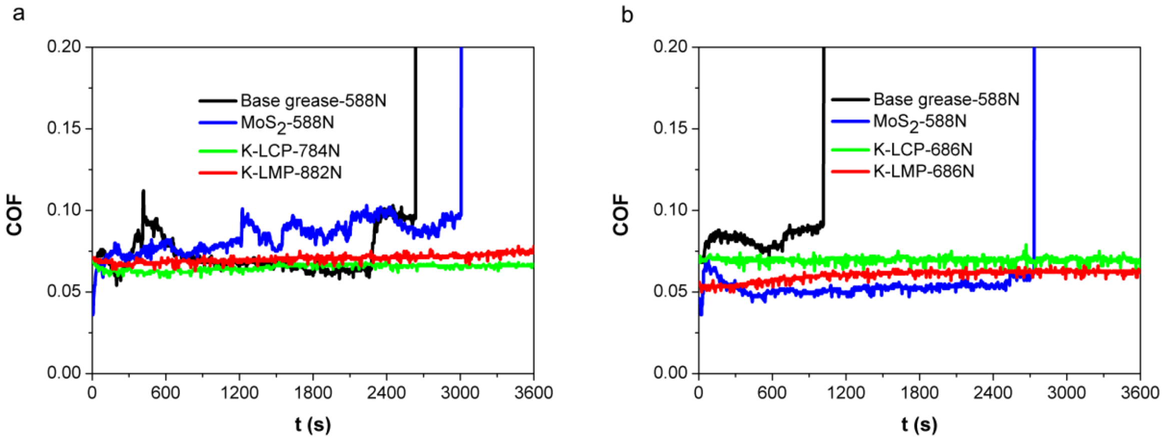

Figure S7. Dynamic friction curves of coconut oil calcium-based grease alone and in the presence of 5.0 % layered magnesium and calcium phosphates under the maximum applied load at different temperatures. (a) 25°C. **(**b) 50°C. Test conditions: 1200 rpm, 3600 s.

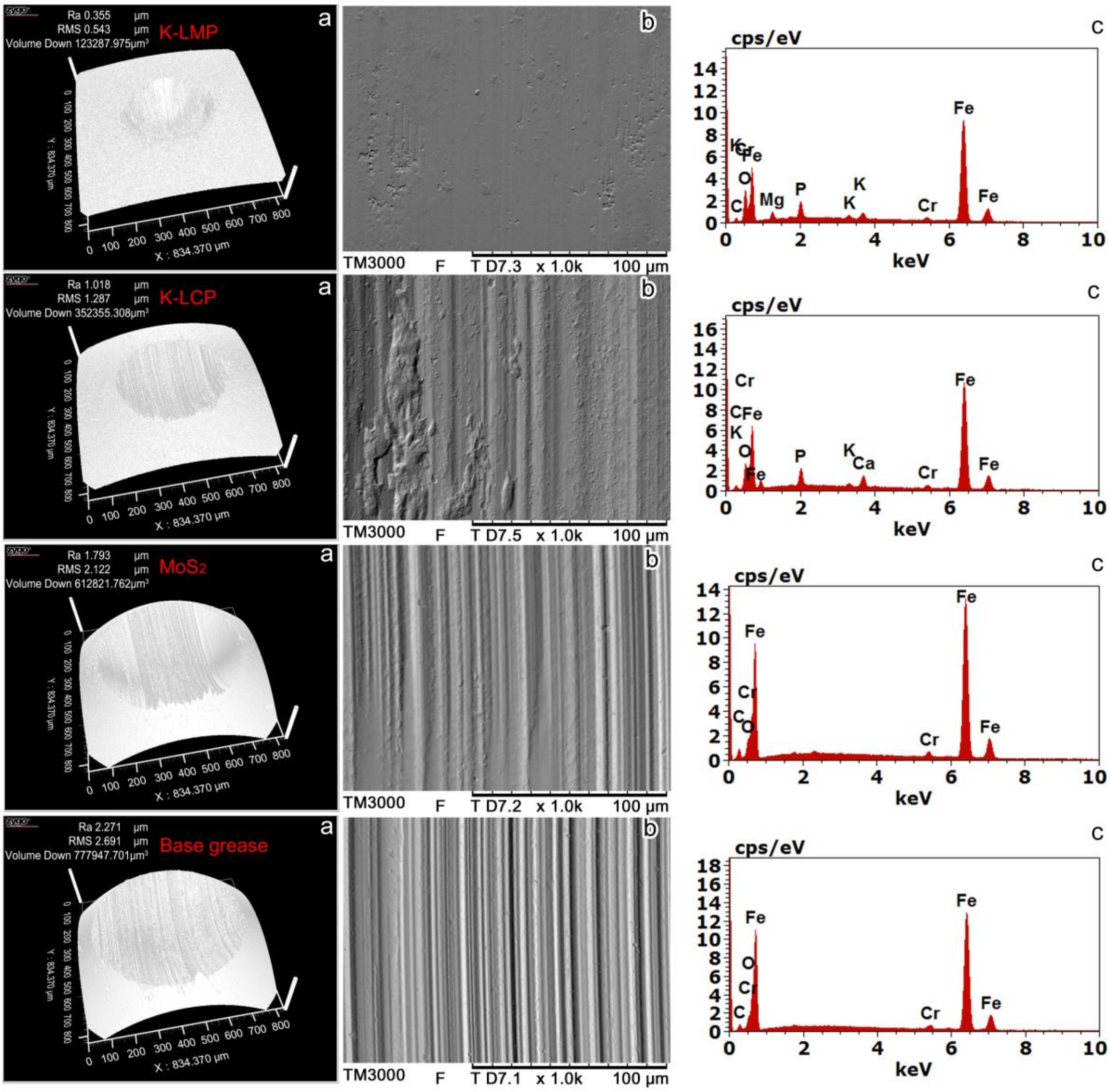

Figure S8. The analysis of the rubbing surface after long run (3600 s) friction-wear test. (a) Non-contact 3D. (b) SEM. (c) EDS. The detail friction-wear test conditions: PAO8 lithium-based grease; 75°C, 1200 rpm, 3600 s, 392 N. In consistent with the results for the coconut oil calcium-based grease, the layered phosphates form protective films on friction pairs during the friction-wear test.

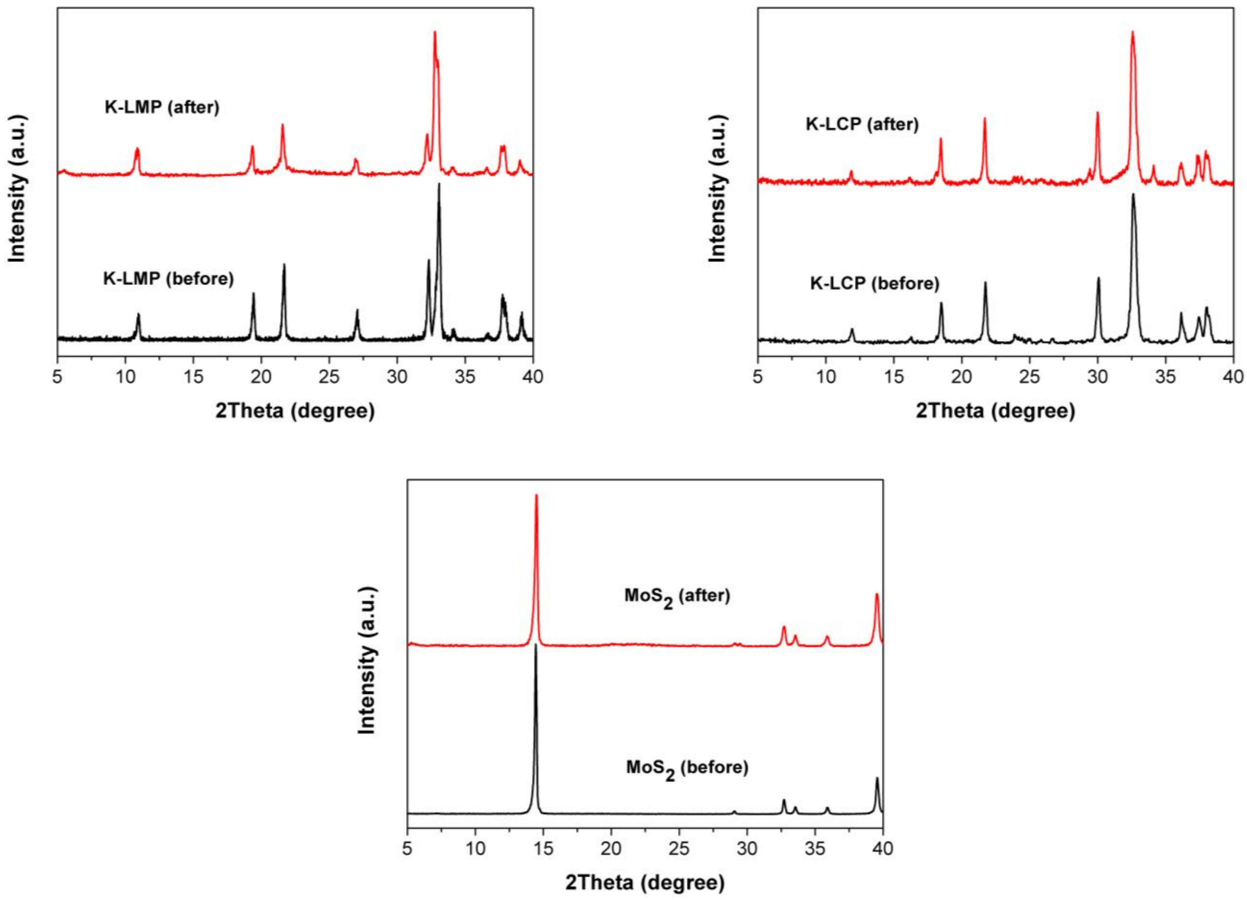

Figure S9. XRD patterns of solid samples before and after friction-wear test.

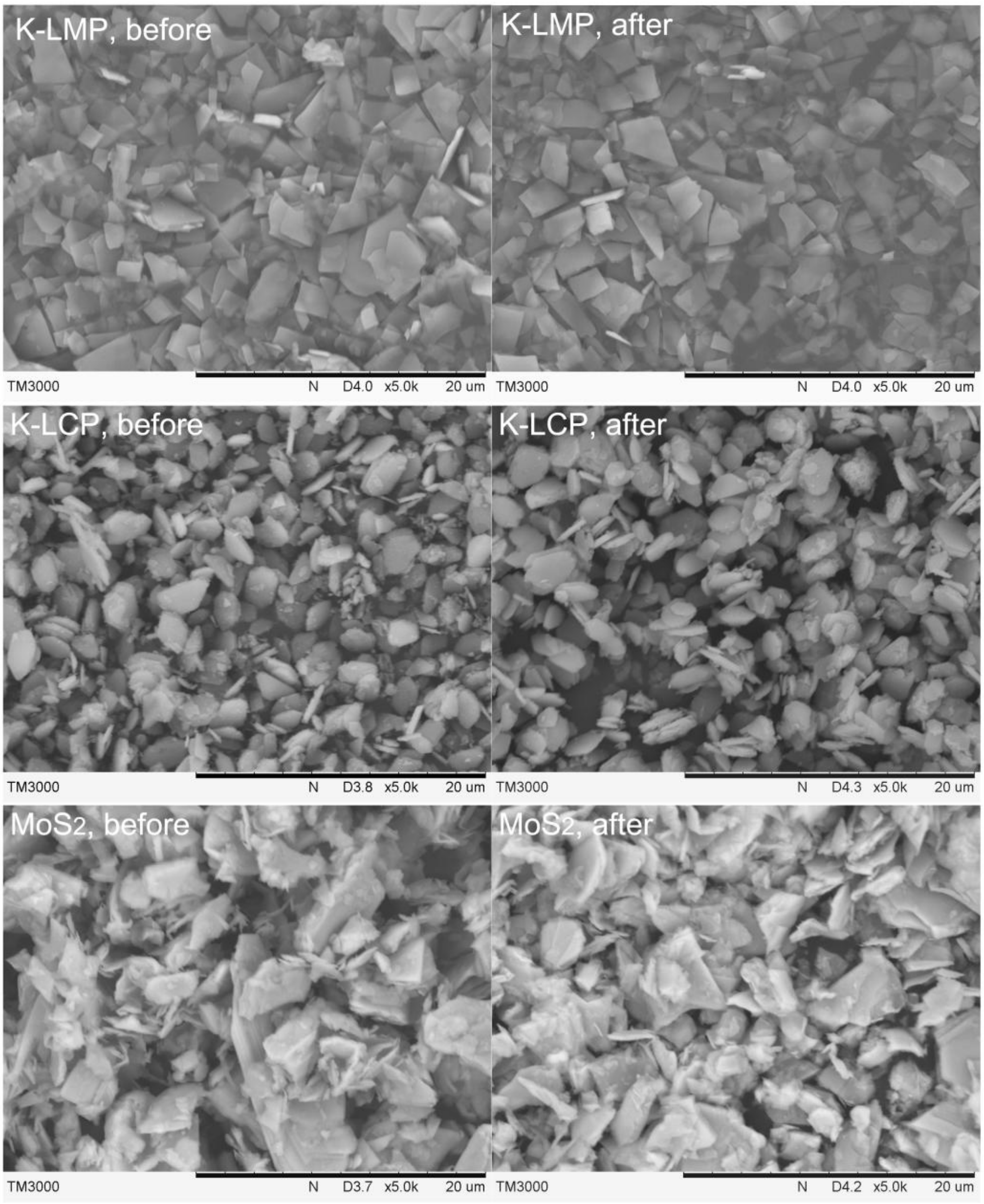

Figure S10. SEM images of solid samples before and after friction-wear test.

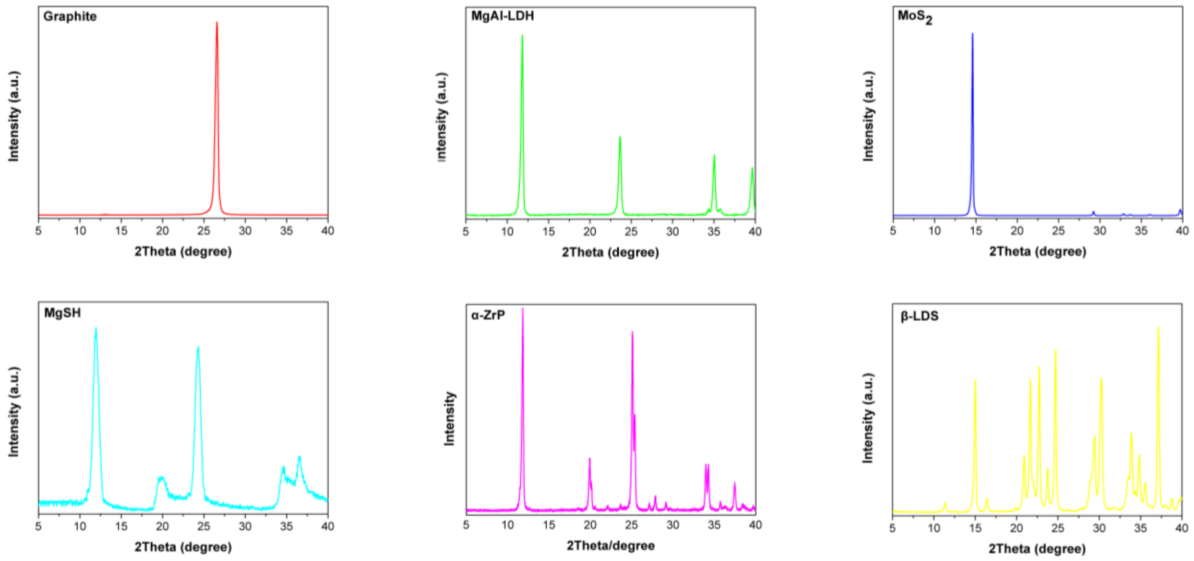

Figure S11. XRD patterns of various typical layered lubricant materials.

Table S4. Typical physicochemical properties of lithium-basesd grease alone and in the presence of 5.0 wt.% β-LDS, α-ZrP, MoS_2, MgSH, MgAl-LDH, Graphite, K-LMP and K-LCP additives.

	Base grease	β-LDS	a-ZrP	MoS_2	MgSH	MgAl-LDH	Graphite	K-LMP	K-LCP	Test standard
Dropping point	205	207	180	200	202	202	204	202	199	ASTM D 566
Cone penetration (0.1 mm)	295	285	285	275	282	291	278	278	276	ASTM D 217
Four-ball wear test WSD (mm)	0.71	0.47	0.39	0.68	0.61	0.53	0.59	0.41	0.48	ASTM D 2266
Four-ball EP test last non-seizure load (N)	353	1568	1235	617	617	490	441	1372	980	ASTM D 2596

Table S5. Typical physicochemical properties of calcium-based grease alone and in the presence of 5.0 wt.% K-LMP, K-LCP and MoS_2 additives.

	Base grease	K-LMP	K-LCP	MoS_2	Test standard
Dropping point	154	140	139	139	ASTM D 566
Cone penetration (0.1 mm)	270	266	268	275	ASTM D 217
Four-ball wear test WSD (mm)	0.68	0.39	0.37	0.61	ASTM D 2266
Four-ball EP test last non-seizure load (N)	510	1235	1235	696	ASTM D 2596
